# Reliability of force plate-based measures of standing balance in the sub-acute stage of post-stroke recovery

**DOI:** 10.1016/j.heliyon.2023.e21046

**Published:** 2023-10-14

**Authors:** Raabeae Aryan, Elizabeth Inness, Kara K. Patterson, George Mochizuki, Avril Mansfield

**Affiliations:** a Rehabilitation Sciences Institute, University of Toronto, Toronto, ON, Canada; b KITE-Toronto Rehabilitation Institute, University Health Network, Toronto, ON, Canada; c School of Kinesiology and Health Science, Faculty of Health, York University, Toronto, ON, Canada; d Evaluative Clinical Sciences, Hurvitz Brain Sciences Program, Sunnybrook Research Institute, Toronto, ON, Canada; e Department of Physical Therapy, University of Toronto, Toronto, ON, Canada

**Keywords:** Stroke, Postural balance, Reliability, Validity, Biomechanics

## Abstract

**Background:**

Difficulty controlling balance is one of the major contributors to the increased risk of falls among individuals with stroke. It is important to use reliable and objective measures to improve examination of balance impairments post-stroke, and to in turn inform clinical decision-making. The main objective of this study was to examine the relative and absolute reliabilities of force plate-based balance measures in quiet standing, in the sub-acute stage of stroke recovery.

**Methods:**

Twenty-four people with sub-acute stroke (mean age = 61 years) performed two trials of quiet standing, each 30 s long. Sixteen force plate-based balance measures in the time, frequency, or nonlinear domains were calculated. Within-session test-retest reliabilities were investigated using intraclass correlation coefficient (ICC), standard error of measurement, and minimal detectable change.

**Results:**

Mean speed of displacements of the centre of pressure along the anterior-posterior axis (ICC = 0.91; CI_95 %_ = [0.83, 0.95]), and directional weight-bearing asymmetry (ICC = 0.91; CI_95 %_ = [0.82, 0.95]) demonstrated high relative reliabilities, followed by the speed-based symmetry index and absolute weight-bearing asymmetry (both ICCs = 0.86; CI_95 %_ = [0.74, 0.93]).

**Conclusions:**

Mean speeds of centre of pressure, directional weight-bearing asymmetry, and speed-based symmetry index are the most reliable force plate-based measures that were evaluated in our study, and can be included in the balance assessments of individuals within the sub-acute stage of post-stroke recovery. These findings can better inform clinicians about the specific balance problems experienced by people in this population.

## 1 Introduction

Poor balance control, while standing or moving, is one of the main contributors to the increased risk of falls among people with stroke [[1], [2], [3]]. Falls can potentially limit an individual's capacity to participate in daily life activities, lead to physical injuries, and can contribute to decreased quality of life [3,4] Thus, precise and objective examination of an individual's balance control is a crucial step for implementing effective post-stroke rehabilitation.

Performance-based balance measures are frequently used to reflect a patient's balance ability [5]. However, it is likely that therapists use their observation of a patient's performance on individual items of these scales, rather than the score itself, to guide clinical decision-making [6,7]. In contrast, instrumented balance assessments, such as force plate-based approaches, are used to provide more objective insights into the specific balance deficits experienced by people with stroke [8]. Force plates can be used to measure fluctuations of the centre of pressure (CoP) under both feet combined when standing still [9]. Two adjacent force plates can also be used to determine the extent of contributions of individual limbs to balance control, synchrony between the lower limbs, and asymmetric weight distribution. These measures are especially important in post-stroke balance assessments, where the asymmetric motor impairments exist [6]. However, despite the promising ability of force plate-based measures in detecting underlying mechanisms of impaired balance control, little is known about their reliability for assessing balance among people with sub-acute stroke.

Although previous studies have reported reliability of force plate-based measures of standing balance in different clinical populations [10,11], only few have reported reliability of these measures in stroke, either in the chronic stage [12,13], or collectively across multiple stages of post-stroke recovery [14,15]. Few studies have established reliability of force plate-based measures of standing balance in the sub-acute stage of stroke recovery. The sub-acute stage of stroke recovery (7 days-6 months post-stroke [16]) is a critical period for neuroplasticity and spontaneous recovery [16], in which patients experience rapid changes in their physiological and functional status. Only Gray et al. (2014) has directly focused on the reliability of some time-domain force plate-based measures of standing balance in sub-acute stroke [17], and found that area of CoP displacements and mean speed of CoP were the two most reliable measures during this post-stroke stage of recovery [17].

Time-domain force plate-based measures can provide clinicians with useful information about asymmetry of balance control, status of between-limbs coordination, and forces required to regulate postural sway [6,[18], [19], [20], [21]]. Alternatively, frequency and nonlinear domains of force plate-based measures can be useful for investigating contributions of different sensory systems to balance control [22,23], and the regularity of CoP time-series [24], respectively. While force plate-based frequency-domain [22] and nonlinear-domain [24] balance measures have been used to detect balance deficits, no previous studies have attempted to establish their reliability in the sub-acute stage of post-stroke recovery.

Given the paucity of research investigating reliability of force plate-based measures of balance control post-stroke, the primary objective of our study was to determine within-session relative and absolute reliabilities of force plate-based time, frequency, and nonlinear-domain measures of quiet standing balance, derived from a single 30 s-long standing trial, in people within the sub-acute stage of stroke recovery. Our main goal was to identify balance measures with high reliability, among those discussed above, as they have the potential to be relevant for addressing post-stroke balance impairments. We hypothesized that our select set of force plate-based measures would demonstrate clinically acceptable levels of relative reliability (ICC>0.8) [25,26] and low measurement error.

## 2 Materials & methods

### 2.1 Participants

This study was a retrospective secondary analysis of the force plate data collected for two other larger projects [27,28]. Both original studies received approvals from the Research Ethics Board of the University Health Network, Toronto, Canada. Participants in the original studies were patients (either post-stroke or acquired brain injury) and healthy adults, who were recruited between August 2013 and January 2017. Written informed consent was obtained from the eligible individuals. Common inclusion criteria of the original studies were: 1) age≥18 years old; 2) ability to stand independently for 1 min; and 3) ability to understand test instructions in English.

Participants were included in the present analysis if they: 1) were individuals with stroke who were receiving or had recently completed inpatient post-stroke rehabilitation at the Toronto Rehabilitation Institute (four participants completed assessments 26–38 days post-discharge from inpatient rehabilitation, while they were still in the sub-acute stage); 2) were in the sub-acute stage of stroke recovery at the time of the force plate assessments; and 3) had two force plate-based assessments of quiet standing balance in one day, each lasting at least 30 s. We excluded participants if they had any condition besides stroke that could potentially affect their balance control (e.g., other neurological conditions, amputations).

Given a power of 80 % and with 2 observations, a sample size of at least 9 participants was required [29] to detect reliability values between ICC = 0.5 (the minimum value that traditionally considered as moderate reliability [30]) and ICC = 0.9 (suggested as demonstrating high reliability in clinical research [25,26]). From a total of 115 participants in the original studies, we included all 24 participants who met criteria of the present study.

### 2.2 Assessment procedures

Participants were instructed to stand in a standard foot position on two adjacent force plates (OR6-7-2000, Advanced Medical Technology Inc., Watertown, Massachusetts, USA), with each foot placed on a separate force plate and oriented 14° outwards, while centres of their heels were 17 cm apart [31]. Participants stood quietly for 30 s in their preferred weight-distributed stance position, with their eyes open, and arms by their sides (if able), while wearing comfortable shoes. Participants who typically wore an ankle-foot orthosis kept their orthosis on during data acquisition. To ensure safety during the force plate-based assessments, participants were supervised by a research assistant and a physiotherapist; however, all participants were able to complete the standing balance tests and maintain the standard foot position, independently and without requiring additional assistance.

Two quiet standing trials were performed in one session (one test trial, one retest trial). The time-intervals between test and retest varied among the participants; however, test and retest trials were not closer than 10 s apart. Participants could request rest breaks if needed during the assessment sessions. Force plate data were sampled at 256 Hz and stored for offline processing. Individuals' height, weight, age, lesion side(s), paretic side, and Berg Balance Scale scores [5] were collected (either directly from participants or from their hospital charts) or assessed by the same research assistant/or physiotherapist. In two cases, where the participants had bilateral stroke lesions, the more-affected side of their body was determined from information in participants’ medical charts and/or performance-based assessments.

### 2.3 Data processing

Forces and moments were filtered using a low-pass 4th-order zero phase-lag Butterworth filter at 10 Hz. CoP under each foot separately, and under both feet combined (net-CoP) were calculated for each trial, along the anterior-posterior (AP) and medial-lateral (ML) directions. CoP time-series’ were down-sampled to 64 Hz; and their means were subtracted from the signals prior to calculating balance measures in the time, frequency, and nonlinear domains. Down-sampling by a factor of 4 allowed us to prepare the CoP time-series for computing our nonlinear balance measure (i.e., sample entropy) and its *a priori* parameters (i.e., segment length, and tolerance radius) [32], while staying above the suggested optimal minimum sampling frequency to calculate the remainder of our force-based balance measures [33]. All measures were computed offline, using a custom-written MATLAB routine (MATLAB and Statistics Toolbox Release 2016b, The MathWorks, Inc., Natick, Massachusetts, USA). To compute the frequency-domain measures, the first 5 s from the CoP time-series’ were removed, and MATLAB Welch's power spectral estimation function (segment length 800 points, with 25 % overlap, frequency resolution of 0.08 Hz) was used. Thus, frequency-domain analysis was performed on the remaining 25 s, on the frequency band of 0–10 Hz. All other measures were calculated from the full 30-s trial.

#### 2.3.1 Time-domain measures: the following measures were selected according to the recommendations regarding the use of force plate-based measures [6,9,34]

a) Root mean square (RMS) of displacements of the net-CoP along the AP and ML axes: RMS of CoP reflects the variability of the time-series [6,35], and is often used as a proxy to quantify amplitude of postural sway [36], since RMS will generally increase as sway increases [6].
b) Mean speeds of net-CoP along the AP, and ML directions: Speed of CoP represents the neuromuscular control exerted by the muscles (primarily in the lower extremities) to respond to postural sway [20,35]; higher speeds can be indicative of a poorer balance control [23,35].
c) The 95 % ellipse area of the net-CoP (both feet combined) was calculated based on the principal component analysis approach [37]. Reportedly, the smaller the ellipse area, the better the general performance of the balance control system [34].
d) Directional, and absolute weight-bearing asymmetries (dir-WBA and abs-WBA): WBA addresses the imbalance in weight distribution between the lower extremities of individuals with hemiparesis while standing in their preferred quite stance posture [6]. Dir-WBA and abs-WBA were calculated as follows [6]: WBA = 100*(*Fz’/Fz*). To yield the dir-WBA, *Fz’* is the mean vertical ground reaction force beneath the paretic limb. To calculate the abs-WBA, *Fz’* is the absolute difference between the mean vertical ground reaction forces under both feet. *Fz* represents the total mean vertical ground reaction force beneath both feet.
e) Symmetry indices were calculated for both the displacement and speed of CoP. We calculated displacement-based and speed-based symmetry indices using the following formula, in which the *RMS* is the RMS of displacement or speed of the AP-CoP under each feet [6,38]: Symmetry Index= (*Non-Paretic RMS)*/(*Non-Paretic RMS* + *Paretic RMS*). Symmetry index is reportedly a clinically useful measure that quantifies individual-limb contributions to balance control [6].
f) Inter-limb cross-correlation reflects how both lower extremities work in synchrony while standing [6], and was calculated by measuring the correlation of displacements of the AP-CoP time series between the paretic and non-paretic extremities in quiet standing [39].

#### 2.3.2 Frequency-domain measures

We selected mean and median power frequencies of the AP and ML components of the net-CoP time-series, since these measures have been previously used to characterize quiet standing balance in stroke and other populations [22,40,41]. Studying the power spectrum of CoP time-series can be useful to demonstrate the relative contributions of different sensory systems to balance control. Higher frequencies indicate faster and smaller balance adjustments [9,35], which are possibly associated with fast corrections in response to sudden instabilities [19] and can be indicators of increased contribution of the somatosensory system to balance control [42].

#### 2.3.3 Nonlinear-domain measures

Sample entropy of CoP is a nonlinear-domain measure which is thought to reflect the attentional demand for balance control [24]; thus, it can highlight the regularity of CoP time-series and complexity of underlying balance control processes. We calculated sample entropy using MATLAB code available on PhysioNet [43]. The net-CoP time-series’ were detrended and normalized by the standard deviation of the signal within the code. According to methods described elsewhere [44], we determined the optimal values for *m* (segment length) and *r* (tolerance radius) as *m* = 2, *r* = 0.03, and *m* = 2, *r* = 0.02 for net AP and ML-CoP displacements, respectively.

### 2.4 Statistical analysis

To detect any bias between repeated measurements, paired t-tests were used to compare force plate-based measures between the test and retest assessments. Bland-Altman plots were generated to visualize biases and agreements between the test and retest trials. Within-session test-retest relative reliability of the force plate-based measures of standing balance was calculated based on a two-way mixed effect ANOVA model to demonstrate agreement between repeated measurements (ICC _2,1_) [30]. ICC values < 0.5, between 0.5 and 0.75, between 0.75 and 0.9, and >0.9 were considered as poor, moderate, good, and excellent relative reliability, respectively [30].

Absolute reliability was determined using the standard error of measurement (SEM), and minimal detectable change (MDC). SEM demonstrates a change in the measurement score which is due to a random error [45]. SEM was calculated using the following formula, where *SD* is the standard deviation of all test and retest assessments [46]: SEM= SD* $\sqrt{1-ICC}$.

MDC is the smallest change in the test score that is not due to a random error and represents a true change [45]. MDC was calculated as follows: MDC = 1.96* $\sqrt{2}$*SEM.

Statistical significance level was considered as α = 0.05. Statistical analysis was completed using the R 3.3.2 (R Core Team, 2017).

## 3 Results

Demographic characteristics of participants are presented in Table 1. The average test-retest time interval was 11.5 min (standard deviation = 8.9 min; range = 10 s to 27.5 min; test-retest intervals for five participants were≤1 min). Mean, standard deviation, and *P*-values of comparison analysis of repeated measurements are presented in Table 2. There were no statistically significant differences between the values of force plate-based measures in test and retest assessments (*P*-value>0.05). Relative (ICC_2,1_ and 95 % confidence intervals, CI) and absolute reliability (SEMs, 95 % MDCs) values are presented in Table 2. Considering the 95 % confidence intervals for each ICC value, mean speed of AP-CoP, and dir-WBA demonstrated good-to-excellent relative reliability (both ICCs = 0.91; 95 % CI = 0.82–0.95). Speed-based symmetry index (ICC = 0.86; 95 % CI = 0.74–0.93), abs-WBA (ICC = 0.86; 95 % CI = 0.74–0.93), and mean speed of ML-CoP (ICC = 0.82; 95 % CI = 0.68–0.91) demonstrated moderate-to-excellent relative reliability. Among the frequency-domain measures, mean power frequency of AP-CoP demonstrated moderate-to-good relative reliability (ICC = 0.79; 95 % CI = 0.62–0.89). For nonlinear-domain measures, sample entropy of AP-CoP also demonstrated moderate-to-good relative reliability (ICC = 0.79; 95 % CI = 0.63–0.89). The lowest relative reliability values, among all categories, were observed for the RMS of AP-CoP (ICC = 0.41) and median power frequency of AP-CoP (ICC = 0.44). Dir-WBA demonstrated high absolute reliability (SEM = 2.53 % body weight, MDC = 7.02 % body weight); while the lowest absolute reliability value was observed for the ellipse area (SEM = 155.7 mm^2^, MDC = 431.6 mm^2^).

**Table 1:** Participant characteristics. Values presented are means with standard deviations in parentheses for continuous variables, and counts for categorical variables.

Participant characteristics	
**Sex** (number)	
Female	6
Male	18
**Age** (years)	61.1 (12.8)
Range	33.0–83.0
**Height** (cm)	171.1 (8.8)
Range	158.0–191.0
**Mass** (kg)	79.0 (16.7)
Range	54.1–116.8
**Stroke type**	
Ischemic	16
Haemorrhagic	7
Unknown	1
**Affected side of the brain** (number)	
Right	10
Left	12
Both	2
**More-affected side of the body** (number)	
Right	12
Left	12
**Time post-stroke** (days)	41.0 (21.9)
Range	12–95
**Berg Balance Scale** (score)	46.3 (11.6)
a Range	6–56

a Out of 24 participants, 16.7 % scored 55–56 on the BBS, 45.8 % scored between 49 and 53, and 37.5 % scored 45 and less, from a maximum of 56 points.

**Table 2:** Means (and standard deviations), *P*-values of paired comparisons of test and retest assessments, and reliability values of force plate-based measures of standing balance, derived from the net-CoP time-series.

Measures	Test	Retest	*P*	ICC_2,1_	CI_95 %_	SEM	MDC
RMS AP-CoP (mm)	6.1 (2.3)	6.7 (2.7)	0.21	0.41	[0.10, 0.65]	1.89	5.23
RMS ML-CoP (mm)	4.0 (1.9)	4.6 (2.2)	0.07	0.74	[0.54, 0.86]	1.05	2.91
95 % Ellipse area (mm^2^)	287 (200)	359 (256)	0.12	0.54	[0.26, 0.74]	155.7	431.6
dir-WBA (body weight%)	45.9 (8.2)	45.3 (8.7)	0.42	0.91	[0.82, 0.95]	2.53	7.02
abs-WBA (body weight%)	13.2 (12.6)	14.5 (13.2)	0.36	0.86	[0.74, 0.93]	4.78	13.3
Inter-limb cross-correlation	0.75 (0.29)	0.76 (0.31)	0.88	0.70	[0.48, 0.84]	0.16	0.45
Symmetry index, displacement	0.54 (0.12)	0.57 (0.11)	0.12	0.70	[0.49, 0.84]	0.06	0.17
Symmetry index, speed	0.59 (0.12)	0.60 (0.11)	0.38	0.86	[0.74, 0.93]	0.04	0.12
Mean speed AP-CoP (mm/s)	14.0 (8.2)	15.1 (10.5)	0.17	0.91	[0.83, 0.95]	2.83	7.84
Mean speed ML-CoP (mm/s)	8.2 (3.2)	8.7 (4.2)	0.24	0.82	[0.68, 0.91]	1.59	4.41

Mean power freq. AP-CoP (Hz)	0.33 (0.19)	0.33 (0.21)	0.95	0.79	[0.62, 0.89]	0.09	0.25
Mean power freq. ML-CoP (Hz)	0.36 (0.14)	0.34 (0.13)	0.26	0.75	[0.55, 0.86]	0.07	0.19
Median power freq. AP-CoP (Hz)	0.23 (0.07)	0.22 (0.10)	0.86	0.44	[0.12, 0.67]	0.06	0.18
Median power freq. ML-CoP (Hz)	0.30 (0.15)	0.28 (0.16)	0.40	0.53	[0.24, 0.73]	0.09	0.26

Sample entropy AP-CoP (bits)	0.96 (0.43)	0.90 (0.48)	0.37	0.79	[0.63, 0.89]	0.21	0.57
Sample entropy ML-CoP (bits)	1.19 (0.37)	1.09 (0.36)	0.06	0.72	[0.50, 0.85]	0.19	0.54

CoP: net centre of pressure. AP: anterior-posterior. ML: medial-lateral. dir-WBA: directional weight-bearing asymmetry. abs-WBA: absolute weight-bearing asymmetry. RMS: root mean square. Freq: frequency. ICC: intraclass correlation coefficient. CI: confidence intervals of ICC. *P*: *P*-value. SEM: standard error of measurement. MDC: 95 % minimal detectable change.

**Fig. 1:**
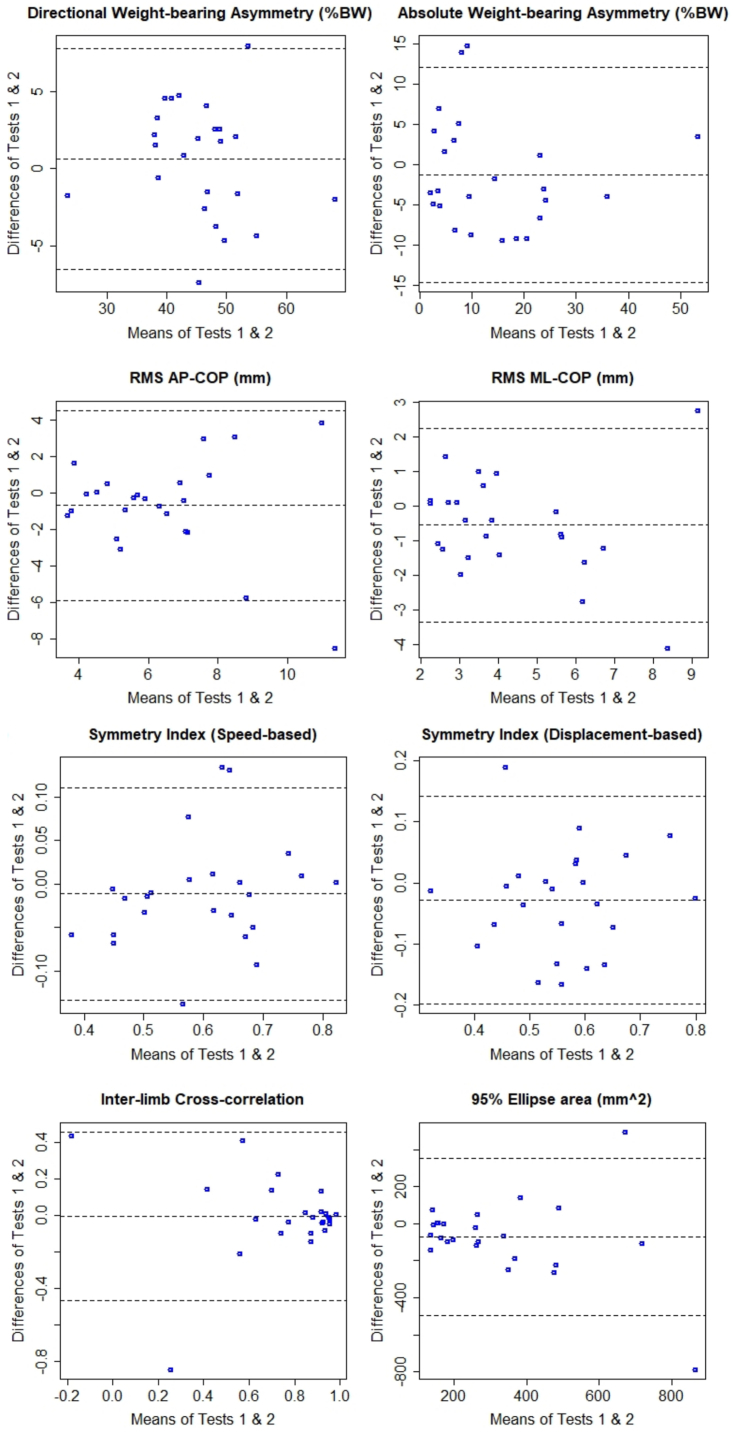
Bland-Altman plots of degrees of agreements between test and retest assessments. Each plot depicts the mean of both test and retest (x-axis) against the difference of test and retest assessment (y-axis). CoP: net centre of pressure. AP: anterior-posterior. ML: medial-lateral. RMS: root mean square. %BW: percentage of body weight.

**Fig. 2:**
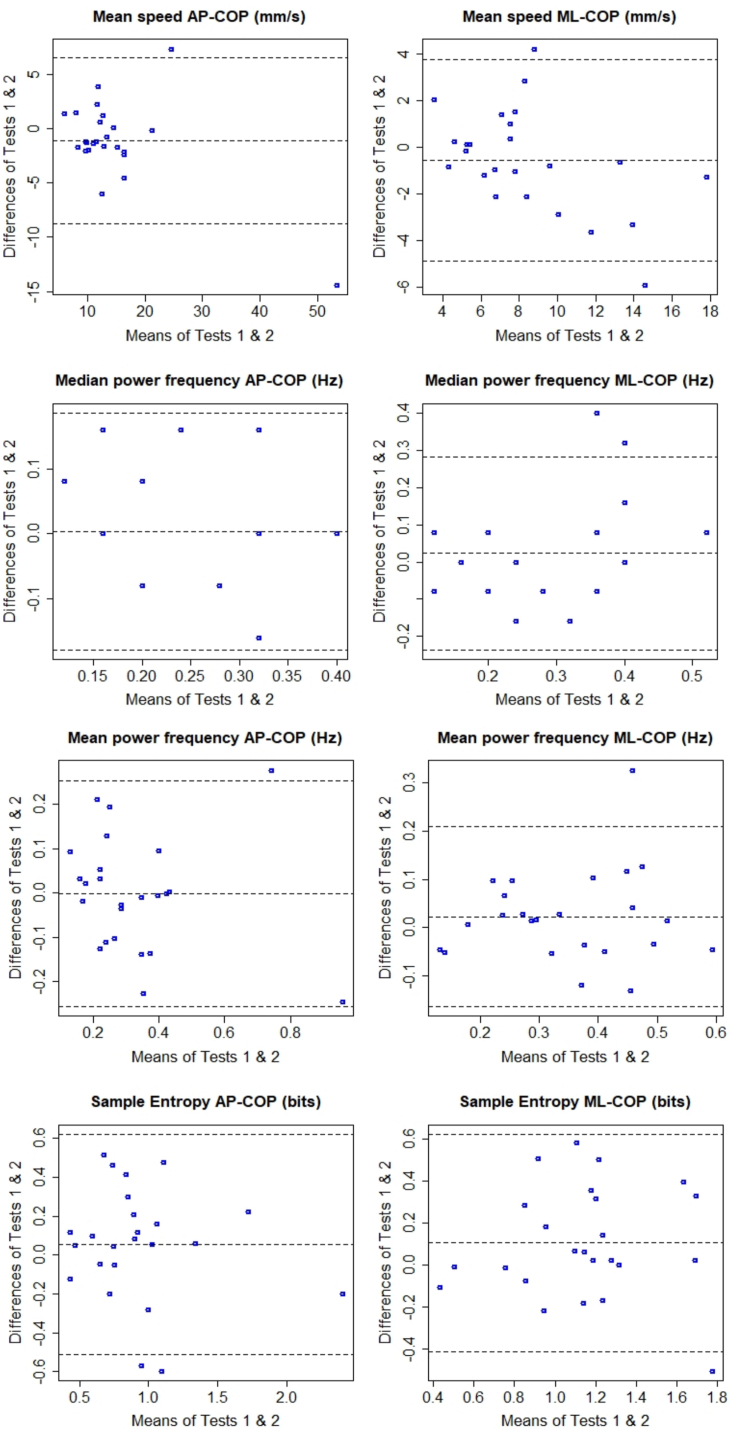
Bland-Altman plots of degrees of agreements between test and retest assessments. Each plot depicts the mean of both test and retest (x-axis) against the difference of test and retest assessment (y-axis). CoP: net centre of pressure. AP: anterior-posterior. ML: medial-lateral.

In each Bland-Altman plot (Fig. 1, Fig. 2), the difference between test and retest (on the y-axis) has been plotted against the mean of test and retest (on the x-axis) for each participant. Zero on the y-axis represents no difference between trials (perfect agreement), the middle dashed line shows the average between-trials difference (average bias), and the other two dashed lines specify ±1.96SD of the average between-trials difference [45]. As shown in Fig. 1, Fig. 2, the majority of average biases are small, and in most cases are very close to zero, except for the 95 % Ellipse area with an average bias of −72 mm^2^. Visual inspection of Fig. 1, Fig. 2 does not suggest existence of systematic biases, since the spreads of scatters around zero seem random and for none of the measures the difference between test and retest trials consistently produces positive, or negative values, particularly for mean speeds of AP-CoP and ML-CoP, dir-WBA, and speed-based symmetry index.

## 4 Discussion

The primary objective of this study was to establish the relative and absolute reliabilities of the force plate-based measures of balance, derived from one trial of 30 s-long quiet standing, in individuals assessed during the sub-acute stage of stroke recovery. Findings of this study demonstrated a range of reliability values across all three categories of force plate-based balance measures. Overall, the mean speed of AP-CoP and dir-WBA showed high relative reliability, followed by the mean speed of ML-CoP and speed-based symmetry index. Dir-WBA also had high absolute reliability for use in the sub-acute stage of stroke recovery.

Speed of CoP has been previously reported as a highly reliable force plate-based balance measure in older adults [47]. Reliability values of the speeds of AP-CoP and ML-CoP in our work are approximately in line with the reliability of the total speed of CoP under the paretic and non-paretic lower limbs in a previous study in sub-acute stroke [17]. This previous study found that speeds of CoP under both paretic and non-paretic limbs had good relative and absolute reliabilities (ICC = 0.82).

Dir-WBA had both high relative reliability and absolute reliability (low measurement error); this finding is in accordance with previous work performed across multiple stages of stroke recovery [15], which reported an excellent ICC estimate value for WBA (ICC≥0.95). Their slightly higher reliability value for WBA can be partly due to using average of multiple trials, and/or including individuals with chronic stroke who may have less variable weight distribution. Abs-WBA, on the other hand, had an ICC estimate value of 0.86, while having high measurement error (high SEM). This high measurement error may have occurred because abs-WBA shows the absolute difference of the proportion of weight distribution between the paretic and non-paretic sides; that is, one-percent change on each lower extremity will be reflected as a two-percent change of the abs-WBA. This can be a possible reason that has amplified the measurement error of the abs-WBA and made it highly variable during test and retest assessments, compared to the dir-WBA. Therefore, use of the dir-WBA is suggested over the abs-WBA, as it has a higher relative reliability (ICC estimate>0.9) and low measurement error. Dir-WBA is also more informative clinically, because it addresses both the direction and magnitude of asymmetric weight-bearing.

We showed that 95 % ellipse area, given the confidence intervals of its ICC, has poor-to-moderate relative reliability (95 % CI = 0.26–0.74), whereas Gasq et al. demonstrated good reliability for this measure (ICC = 0.76) [14]. This difference can be in part due to Gasq et al. using a longer CoP signal than us (approximately 51 s), and taking the average of multiple trials. It has been shown that reliability of force plate measures is improved when the sampling duration is increased and/or when multiple trials are averaged [48]. Moreover, in our study, ellipse area had high measurement error (48 % of its mean). Again, this emphasizes the high within-subjects variations of the ellipse area calculated from the net-CoP time-series; this is likely due to the lack of sufficient control of the CoP under the paretic side and its effects on the net-CoP values.

We found poor-to-good relative reliability (given the ICC confidence intervals) for the displacement-based symmetry index, whereas the speed-based symmetry index showed moderate-to-excellent relative reliability. These results are in accordance with another study in chronic stroke, in which a slightly higher reliability was shown for speed-based symmetry index than the displacement-based index [13]. It is speculated that the displacement-based symmetry index shows lower relative reliability than the speed-based index because the RMS of AP-CoP (which is used to calculate this measure) has poorer reliability than the RMS of speed of AP-CoP [48,49]. As such, if clinicians are interested in using the symmetry index to evaluate the contributions of individual-limbs to balance control in sub-acute stroke, using the speed-based symmetry index that has a lower measurement error and better relative reliability is suggested over the displacement-based symmetry index.

RMS of displacements of CoP along the AP and ML axes, and inter-limb cross-correlation demonstrated poor-to-moderate relative reliabilities in the present study. Although current findings for RMS of displacements of CoP are in accordance with the previous works in healthy young [40] and older adults [47], they do not agree with the findings of another study in chronic stroke where a greater reliability was shown for RMS of displacements of CoP (ICC = 0.91) [13]. This finding emphasizes the importance of determining reliability of measures for every distinct population of interest. Low reliability of RMS of CoP, inter-limb cross-correlation, and ellipse area suggest that they might not be appropriate choices for characterizing balance in the sub-acute stage of stroke recovery.

In general, the frequency-domain measures included in our study demonstrated poor-to-good reliability. This generally lower reliability of frequency-domain measures than the other force plate-based measures can be due in part to the short sampling duration of the CoP time-series [40]. In clinics, it is feasible to capture the CoP time-series in trials as short as 30 s. However, the frequency content of the signals sampled over shorter durations may differ from those with longer durations [40]. Thus, the longer a signal, the more likely it is to capture low frequency events. A longer data acquisition period might improve the reliability of frequency-domain force plate-based measures. Previous work in healthy older adults showed that to obtain an ICC≥0.9 for the median power frequency of AP-CoP and ML-CoP, respectively, 20 and 13 repetitions of 30 s-long trials must be averaged [47]. Thus, this solution seems less feasible in clinical settings, since fatigue due to the lengthy tests can negatively impact the assessment results, and the time dedicated to multiple administrations of this test can be spent on testing other aspects of balance control and progressing toward testing more challenging tasks.

Our findings showed that sample entropy of displacements of both AP-CoP and ML-CoP had moderate-to-good relative reliabilities, with a slightly higher measurement error for sample entropy of AP-CoP than ML-CoP. A similar but slightly higher ICC estimate was observed in chronic stroke for sample entropy calculated from the resultant CoP time-series (ICC_3,1_ = 0.80) [13]. Similar to the frequency-domain measures, the length of the force plate signal is one of the factors that affect the values of sample entropy of CoP [50]. Using trials longer than 1 min has been recommended for computing sample entropy values [50].

It has been recommended that, for the purpose of decision-making in medical and sport sciences, measures with reliability values above 0.9 should be considered as having high reliability, between 0.9 and 0.8 as having moderate reliability, and less than 0.8 as having insufficient reliability [25,26]. Therefore, our study suggests that dir-WBA, mean speeds of AP and ML-CoP, and speed-based symmetry index can be considered as reliable force plate-based measures for clinical evaluation of balance in individuals with sub-acute stroke, since all have both relative reliability values greater than 0.8, and low measurement errors.

This study was not without limitations. We used 30 s-long time-series to estimate the reliability of force plate-based measures in quiet standing with eyes open. Therefore, our findings might not apply to situations where individuals are unable to stand for at least 30 s, to other variations of standing such as standing on unstable surfaces or moving platforms and reactive balance tests with internal and external perturbations, or balance assessments administered in sitting or with eyes-closed. We conducted this study in a clinical/research laboratory; thus, our findings might not be applicable to tests performed in the other settings (e.g., at home, gym). Our reliability values are within-session test-retest reliability estimates; however, it is likely that clinicians evaluate balance control over consecutive days, rather than multiple times within a session, thus establishing between-session reliability of force plate-based measures can be more clinically useful. We identified the reliability values in the sub-acute stage of stroke recovery; therefore, the findings might not be generalizable to the chronic stroke, or to other neurological conditions.

## 5 Conclusions

Our findings suggest that the directional weight-bearing asymmetry, mean speeds of AP and ML-CoP, and speed-based symmetry index, calculated from only one 30 s upright standing trial, are reliable force plate-based measures for balance assessments of individuals within the sub-acute stage of stroke recovery. These reliable measures can potentially be used to objectively detect specific balance problems experienced by people in this population. However, to improve their clinical relevance, future research is required to determine the validity of these measures through identifying their correlation with risk of falls and performance-based balance measures, and to explore their association with other performance-based mobility measures. Better balance assessments using objective and reliable measures will lead to better understanding of the extent of balance impairments and may, in turn, results in better rehabilitation interventions.

## Ethics statement

This study was a secondary analysis of data collected for two larger trials; both original trials received approvals from the Research Ethics Board of the University Health Network, Toronto, Canada (ethics approval numbers: 13–6669 and 14–8085). Written informed consent was obtained from all the eligible individuals.

## Funding statement

Equipment and space have been funded with grants from the Canada Foundation for Innovation, 10.13039/100008185Ontario Innovation Trust, and the 10.13039/501100015668Ministry of Research and Innovation. Avril Mansfield was supported by a New Investigator Award from the 10.13039/501100000024Canadian Institutes of Health Research (MSH-141983). Raabeae Aryan was supported by the Peterborough K.M. Hunter Charitable Foundation Graduate Award, 10.13039/501100002202Toronto Rehabilitation Institute Student Scholarship, QEII/Heart and Stroke Foundation of Ontario Graduate Scholarship in Science and Technology, Unilever/Lipton Graduate Fellowships in Neurosciences, and Rehabilitation Sciences Institute-10.13039/501100003579University of Toronto Doctoral Completion Award. The authors confirm that the funders had no influence over the study design, data collection, analysis and interpretation of data, writing of the report, and the decision to submit the article for publication.

## Data statement

The participants of this study did not give written consent for their data to be shared publicly; therefore, supporting/raw data is not available.

## CRediT authorship contribution statement

**Raabeae Aryan:** Conceptualization, Formal analysis, Methodology, Writing – original draft, Writing – review & editing. **Elizabeth Inness:** Funding acquisition, Resources, Writing – review & editing, Data curation, Supervision. **Kara K. Patterson:** Methodology, Writing – review & editing, Supervision. **George Mochizuki:** Methodology, Writing – review & editing, Supervision. **Avril Mansfield:** Conceptualization, Data curation, Funding acquisition, Methodology, Resources, Supervision, Writing – review & editing.

## Declaration of competing interest

The authors declare that they have no known competing financial interests or personal relationships that could have appeared to influence the work reported in this paper.
